# Impact of Perceived Barriers on Patient Engagement and Attitudes towards Transition and Transfer

**DOI:** 10.3390/children9091273

**Published:** 2022-08-24

**Authors:** Megan Drovetta, Emily Cramer, Alaina Linafelter, Jordan Sevart, Michele Maddux

**Affiliations:** 1 School of Social Welfare, The University of Kansas, Lawrence, Kansas City, MO 66045, USA; 2 Health Services and Outcomes Research, Children’s Mercy Kansas City, Kansas City, MO 64108, USA; 3 Department of Pediatrics, School of Medicine, University of Missouri Kansas City, Kansas City, MO 64110, USA; 4 Division of Gastroenterology, Children’s Mercy Kansas City, Kansas City, MO 64108, USA; 5 Division of Developmental and Behavioral Health, Children’s Mercy Kansas City, Kansas City, MO 64108, USA

**Keywords:** young adults, transition, barriers, treatment responsibility

## Abstract

Objective: This study is a preliminary evaluation of how perceived barriers towards transition might impact patient attitudes towards their own readiness and ability to transition, self-efficacy towards their IBD, and the allocation of treatment responsibility. Methods: A sample of 81 young adults with IBD were seen for standard care in a Young Adult Clinic (YAC). Patients completed questionnaires on perceived transition barriers; perceived confidence, importance, motivation, and readiness towards transition and transfer; IBD self-efficacy; and allocation of treatment responsibility. Path model analyses were conducted. Results: Not knowing how and who to transfer to and not understanding insurance details were the most commonly endorsed perceived barriers to transition. A significant relationship was found between the attitude toward transition and allocation of treatment responsibility, but no meaningful indirect effects were found from perceived barriers to the allocation of treatment responsibility, using attitudes toward transition as an intervening variable. The relationship between perceived barriers and allocation of treatment responsibility was at least partially explained by examining the intervening effects of attitudes toward transfer and self-efficacy. Conclusions: The study findings carry important implications for targets of clinical intervention to assist young adults with IBD in engaging in their health care and ultimately transferring into adult care.

## 1. Introduction

The Society for Adolescent Medicine defines transition to adult medical care as the “purposeful, planned movement of adolescents and young adults with chronic physical and medical conditions from child-centered to adult-oriented health-care systems” [1]. It refers to two closely related processes—transition and transfer. During the transition process, disease-related responsibilities are gradually re-allocated between adolescents and caregivers, and the responsibility for managing health care demands (e.g., taking medications and scheduling appointments) shifts from the caregiver to the young adult. During the transfer process, youth move from their pediatric providers to an adult provider who will assume the primary responsibility for the youth’s medical needs thereafter.

Transition into young adulthood and transfer to adult health care can be daunting for many youths. This is typically a period during which youth are taking on greater responsibility and ownership for their health care needs and making plans to leave their pediatric care teams for adult ones. Because of the challenges involved in these processes, both in the US and other continents (e.g., Europe), processes for purposeful transitions have been mandated by several professional organizations, including the American Academy of Pediatrics [2] and the European Academy of Paediatrics [3].

Transition is highly relevant for youth with inflammatory bowel disease (IBD), a disease characterized by chronic inflammation of the intestines, particularly as it has peak incidence in adolescence. A national survey of adult gastroenterologists revealed that despite being essential patient competencies for a successful transfer of care, 55% and 69% of gastroenterologists reported that young adults with IBD demonstrate deficits in knowledge of their medical history and medication regimens, respectively [4]. Further research suggests that many youth with IBD inaccurately identify their health care resources (e.g., insurance provider and pharmacy name) and pertinent details of their medical history (e.g., diagnosis and surgeries/procedures) [5], few adolescents manage their illness/treatment independently [6,7], most lack the knowledge and skills to make appropriate health decisions and maintain health-promoting behaviors, and few understand health insurance basics and the differences between pediatric and adult services [7,8,9,10]. These issues are not unique to pediatric IBD, are shared by other pediatric patient populations [11], and have prompted various pediatric-to-adult care transition models for youth with pediatric-onset chronic conditions [12]. The importance of preparing chronically ill adolescents to manage their health is widely recognized. Yet, the National Survey of Children with Special Health Care Needs estimates that only 40% of youth receive support to effectively transition [13]. In addition, among pediatric centers offering transition support, patient-reported outcomes data are not routinely collected as part of standard care, thereby limiting opportunities to guide transition-focused intervention or evaluate changes in patient functioning and transition preparedness over time.

Several theoretical models are salient to the process of transition. One of those is Social-ecological Model of AYA (adolescents and young adults) Readiness for Transition (SMART), as it identifies modifiable patient variables that are significant predictors of transition readiness and key for successful transfer to adult care [14]. The Stages of Change model also posits that health decisions and behaviors are affected by where a patient is in the change process, and can be useful to identify appropriate interventions to foster positive behavior change. As one example, the more ready a patient is to engage in a behavior change, the more likely this will translate into a behavior [15]. Relatedly, two major factors that have been found to affect a person’s readiness to change are importance and self-efficacy, important tenants of motivational interviewing. These have been linked to greater engagement in health care interventions and patient outcomes [16,17,18].

As such, we aimed to evaluate associations between various patient-reported outcomes, such as perceived barriers and attitudes towards transition, self-efficacy, and the allocation of treatment responsibly among young adults with IBD receiving standard care in a multidisciplinary transition clinic, the Young Adult Clinic (YAC). As the impact of perceived barriers towards transition has not previously been evaluated, we also took an exploratory approach to evaluate how perceived barriers towards transition might impact patient attitudes towards their own readiness and ability to transition, self-efficacy towards their IBD, and the responsible allocation of treatment.

## 2. Materials and Methods

### 2.1. Participants and Procedures

Eighty-nine young adults with IBD were seen in the YAC, which targets transition planning among young adults ages 17 and older. This clinic takes place in the Gastroenterology Clinic at a children’s hospital in the Midwest, whose primary service area covers a 150-county area comprised or urban and rural populations in two states, 57% of which are White. As part of standard care, patients completed questionnaires on perceived transition barriers; perceived confidence, importance, motivation, and readiness towards transition and transfer; IBD self-efficacy; and the allocation of treatment responsibility. This information was reviewed by the medical team and used to guide the content of the visit. Of the 89 young adults seen in YAC, eight (9%) had incomplete questionnaires and were excluded from analyses; as such, the final sample described herein included 81 young adults. Details on the development of this clinic have previously been published [19]. Approval for this retrospective chart review was obtained from the appropriate institutional review board.

Psychosocial questionnaires were sent electronically to patients approximately 2–3 weeks before a scheduled visit and they were completed via REDCap. REDCap (Research Electronic Data Capture) is a secure, web-based application designed to support data capture for research studies, providing (1) an intuitive interface for validated data entry, (2) audit trails for tracking data manipulation and export procedures, (3) automated export procedures for seamless data downloads to common statistical packages, and (4) procedures for importing data from external sources [20]. Patients were sent a REDCap-generated email link to the clinic questionnaires with instructions about completing them. Two waves of reminder emails automatically generated within REDCap were sent to patients after the initial email invitation was sent. All data were incorporated into the article.

### 2.2. Measure

*Transition Ratings:* On a scale from 0 (not at all) to 10 (very much), patients were asked to rate the following: (1) How confident they are in their ability to successfully take on more responsibilities for their IBD and treatment. (2) How motivated they are to take on more responsibilities for their IBD and treatment. (3) How important they think it is to take on more responsibilities for their IBD and treatment. (4) How ready they feel to take on more responsibilities for their IBD and treatment. All study measures were assessed for internal consistency using Cronbach’s alpha. The internal consistency for this sample was high (Cronbach’s α = 0.89).

*Transfer Ratings:* On a scale from 0 (not at all) to 10 (very much), and patients were asked to rate the following: (1) How confident they are in their ability to successfully transfer to an adult doctor. (2) How motivated they are to successfully transfer to an adult doctor. (3) How important they think it is to successfully transfer to an adult doctor. (4) How ready they feel to successfully transfer to an adult doctor. Internal consistency for this sample was high (Cronbach’s α = 0.89). Total scores for attitudes toward transition and transfer were calculated as the mean of the four items: confidence, motivation, importance, and readiness. Of note, instructions for transition and transfer ratings included a definition of transition and transfer, according to definitions published by the American Academy of Pediatrics. That is, “Transition is when you take on more responsibilities for your disease and treatment. Transfer is when you move on to an adult doctor to take over your medical care”.

*Barriers Assessment:* Patients were asked to select from a list of 12 barriers that were perceived to make it difficult for them to transition into adult care successfully. These include not knowing how to manage health needs, not knowing one’s medical history, not understanding insurance costs/plan/coverage details, and not knowing how/who to transfer to. Patients were instructed to select all that applied. This list of barriers was created based on patient and caregiver reports during routine clinic visits and a review of the relevant literature (e.g., [21,22]).

*Allocation of Treatment Responsibility (ATR)* [23]: ATR is an 18-item measure for use in pediatrics, designed to assess who is responsible for various treatment regimen-related tasks for caregivers and patients. The measure has three subscales to assess responsibility for oral medication, clinic visits, and laboratory visits, and provides total scores for both patient responsibility and caregiver responsibility. Respondents rated themselves on their own level of treatment responsibility for each task and then rate the caregiver’s responsibility for each task, with higher scores denoting a higher responsibility. Patient-report forms demonstrated good reliability (α = 0.91 for patient). ATR was originally developed for pediatric transplant patients but has been adapted for use with other pediatric conditions [24]. Internal consistency for patient responsibility for this sample was high (Cronbach’s α = 0.91).

*IBD Self-Efficacy Scale (IBD-SES)* [25]: The IBD-SES is a 29-item disease-specific measure of self-efficacy. The measure evaluates self-efficacy in four domains, including managing stress and emotions, managing medical care, managing symptoms and disease, and maintaining remission. Respondents rated each item on a 10-point Likert scale, with high scores denoting a higher self-efficacy. The internal consistency for this sample was high (Cronbach’s α = 0.70).

### 2.3. Statistical Analyses

**Figure 1:**
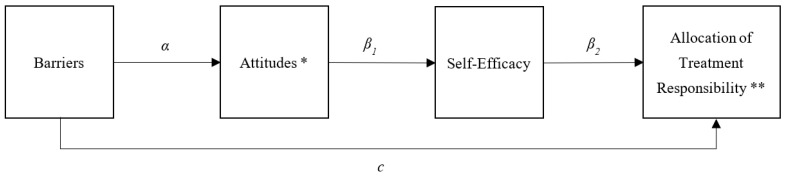
Path model of the relationship between barriers and the allocation of treatment responsibility. Note: the hypothesized model tests the indirect effect of barriers on patient-reported allocation of treatment responsibility through two sequential mediator variables. The indirect effect is calculated as αβ1β2. * Separate path models were estimated for attitudes toward transfer and attitudes toward transition. ** Allocation of treatment responsibility is treated as three separate outcome variables: ATR medications, ATR appointments, and ATR labs. All three outcomes are included simultaneously in the path models.

Descriptive statistics were used to summarize patient demographic characteristics as well as patient-reported questionnaire data. Bivariate correlations were conducted to identify significant relationships between patient-reported outcome variables. Descriptive analyses were conducted in SPSS 24.0 (SPSS Inc., Chicago, IL, USA). Linear regression path models were used to explore the direct and indirect relationships between perceived barriers, attitudes towards transition/transfer, and self-efficacy in predicting patient-reported allocation of treatment responsibility. Total scores for attitudes toward transition and transfer and self-efficacy were treated as intervening variables in the relationship between perceived barriers and the allocation of treatment responsibility (see Figure 1). Because attitudes toward transition and attitudes toward transfer are distinct concepts, measured with different scales, separate path models were fit for transition and transfer. Three allocation of treatment responsibility (ATR) outcomes were modeled simultaneously: medications, appointments, and labs. Indirect effects were calculated as the product of three regression paths: barriers to attitudes (α), attitudes on self-efficacy (β_1_), and self-efficacy on the allocation of treatment responsibility (β_2_). Path models were analyzed using Mplus version 8 [26]. Direct and indirect effects were assessed using 95% confidence intervals constructed from 5000 samples drawn using a bias-corrected bootstrapping approach.

To determine whether caregivers or adolescents were more responsible for a given IBD-related task, based on patient report, we modified the scoring method for the ATR in a manner previously done with this measure [27]. Scores were obtained by subtracting the patient ratings of their responsibility from patient ratings of the caregiver responsibility. Negative values indicate that the adolescent is primarily responsible for the task, while positive values indicate that the caregiver is primarily responsible, and values close to 0 indicate equal responsibility for the task.

## 3. Results

### Participant and Practice Characteristics

The data presented represent data collected as part of standard care and include young adults with IBD (*n* = 81), ages 17–22 (*M* = 18.84, *SD* = 1.26) seen in YAC. Here, 41 patients were male (50.6%) and almost 72% of patients were Caucasian. Most patients seen were diagnosed with Crohn’s disease (80.2%). The insurance status was as follows: 70.4% of patients had private insurance, 24.7% had Medicaid, and 4.9% were self-pay (Table 1).

**Figure 2:**
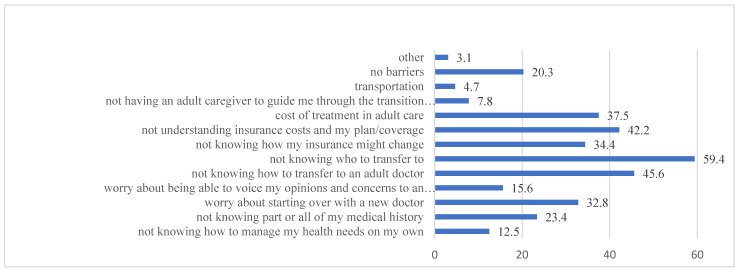
Summary of the transition and transfer barriers endorsed. Note: the *x*-axis corresponds to the number of patients (%).

Average patient-report ratings of confidence, motivation, importance, and readiness towards transition (i.e., taking on more responsibilities for their IBD and treatment) and transfer (i.e., ability to successfully transfer to an adult doctor) were high (Table 2). Approximately 20% of patients endorsed low to moderate transition ratings and approximately 25% of patients endorsed low to moderate transfer ratings. Not knowing how and who to transfer to and not understanding insurance costs/plan/coverage were the most endorsed perceived barriers to transition (Figure 2). On average, out of the 12 total barriers, patients endorsed 3.44 barriers (*SD* = 2.21), with 25.9% of patients endorsing five or more barriers. All patient-reported ATR scales yielded negative values, indicating that patients reported being more responsible for their IBD medications, appointments, and labs compared with their caregivers. The total scores for each scale were: oral medication (*M* = −6.35, *SD* = 9.60), labs (*M* = −0.32, *SD* = 5.82), appointments (*M* = −0.55, *SD* = 11.71), and total (*M* = −9.51, *SD* = 24.29). Patient responsibility for oral medication was the highest, followed by responsibility for clinic appointments and labs. The average patient-report ratings of IBD self-efficacy were also high (*M* = 52.5, *SD* = 6.8).

No substantial correlations with insurance were found and no differences in study variables by diagnosis, insurance, or ethnicity were found. There were no differences in insurance-specific barriers by insurance type. Males were more likely to report caregivers taking greater responsibility for their overall health and treatment (t (75) = −0.386, *p* = 0.035.

Several noteworthy correlations were found (Table 3). Increasing patient age was associated with greater patient responsibility for their IBD (r = −0.51, *p* = 0.000), greater transition importance (r = 0.31, *p* = 0.005), and greater transition readiness (r = 0.30, *p* = 0.007). Greater perceived barriers were associated with decreased confidence (r = −0.52, *p* = 0.000), motivation (r = −0.36, *p* = 0.001), and readiness (r = −0.38, *p* = 0.000) towards transfer. Greater IBD self-efficacy was associated with greater confidence (r = 0.23, *p* = 0.038), importance (r = 0.38, *p* = 0.001), and readiness (r = 0.26, *p* = 0.018) towards transfer. Greater patient responsibility for their IBD was associated with greater attitudes towards transition across all four domains (confidence (r = −0.49, *p* = 0.000), motivation (r = −0.36, *p* = 0.002), importance (r = −0.36, *p* = 0.002), and readiness (r = −0.44, *p* = 0.000) as well as greater attitudes towards transfer across all four domains (confidence (r = −0.34, *p* = 0.003), motivation (r = −0.28, *p* = 0.015), importance (r = −0.24, *p* = 0.043), and readiness (r = −0.32, *p* = 0.006)).

Path model analyses were conducted to evaluate the relationship between perceived barriers, patient attitudes towards transition, and patient engagement in their treatment. In Model 1, more positive attitudes toward transition were related to higher levels of self-efficacy (β = 0.38; CI = 0.18–0.55), and increased patient responsibility for their oral medications (β = −0.43; CI = −0.61–−0.23), appointments (β = −0.40; CI = −0.60–−0.20), and labs (β = −0.36; CI = −0.57–−0.15) (see Table 4). There were no meaningful indirect effects from perceived barriers to allocation of treatment responsibility using attitudes toward transition as an intervening variable.

In Model 2 (Table 5), a higher number of perceived barriers were associated with a less positive attitude toward transfer (β = −0.41; CI = −0.60–−0.18). More positive attitudes toward transfer were related to higher levels of self-efficacy (β = 0.26; CI = 0.04–0.46), and increased patient responsibility for their oral medications (β = −0.37; CI = −0.63–−0.13), appointments (β = −0.29; CI = −0.55–−0.01), and labs (β = −0.25; CI = −0.47–−0.02). Furthermore, the relationship between perceived barriers and the allocation of treatment responsibility was at least partially explained by examining the intervening effects of attitudes toward transfer and self-efficacy. The total indirect effects of perceived barriers on patient responsibility for oral medications (β = 0.18; ES = 0.07; ES CI = 0.02–0.14) and appointments (β = 0.14; ES = 0.13; ES CI = 0.04–0.28) were positive and the confidence interval excluded 0. The total indirect effect of perceived barriers on patient responsibility for labs (β = 0.13; ES = 0.01; ES CI = 0.00–0.03) was positive, but the confidence interval included 0. The total indirect effect was calculated as the product of multiple paths.

## 4. Discussion

Little is known about patient perceived barriers towards successfully taking on more responsibility for their IBD and treatment (i.e., transition) or towards successfully transferring to an adult doctor (i.e., transfer), and its potential impact on patient attitudes and engagement. This study is aimed at evaluating patient-reported outcomes such as perceived barriers towards transition, self-efficacy, and the allocation of treatment responsibility among young adults with IBD receiving standard care in a young adult clinic. We also took an exploratory approach to evaluate how perceived barriers towards transition might impact patient attitudes towards their own readiness and ability to transition and transfer to adult care systems.

Overall, study findings suggest that when patients perceive greater barriers towards their ability to transfer to adult care, they experience more negative attitudes towards the transfer process, which in turn leads to lower levels of self-efficacy towards their disease and treatment. This, in turn, leads to a lower level of patient responsibility for their treatment across medication, clinic visits, and labs. In addition, the relationship between perceived barriers and treatment responsibility is partially explained by a patient’s attitudes toward transfer and their level of self-efficacy towards their IBD and treatment. This suggests that there are multiple modifiable treatment targets that might ultimately impact patient engagement in their health care. Clinical intervention can then be tailored to the unique obstacles and challenges perceived by young adults, guide problem-solving and skills development, and an overall smoother transfer process may then occur. For example, if patients identify not knowing how to transfer and who to transfer to as a barrier, intervention might target the identification of adult GI providers (from an existing list, if available, or the Doc4Me© app (The NASPGHAN Foundation for Children’s Digestive Health and Nutrition 2017, Ambler PA) and educating patients on the process taken by the pediatric clinic to transfer medical records and collaborate with the receiving adult provider. Education can also be provided on important questions to ask an adult gastroenterologist, which are usually tailored to the patient’s specific treatment regimen, insurance coverage, or other factors. As another example, insurance-related barriers might be addressed by connecting patients with a social worker or financial counselor to educate patients on insurance basics and explore insurance coverage (as needed). The IBD Transfer Toolkit is one tool that can be used by pediatric GI providers to educate and guide patients through the transfer process [28]. Summarizing transition-focused clinical interventions is beyond the scope of this paper and there are several noteworthy review articles that do so [12,29,30].

Extant literature is limited to the identification of transition barriers from patient, parent, and provider stakeholders [21,22], and there are no prior studies on the impact of perceived transition barriers on patient outcomes. Although not a direct comparison, prior studies on barriers to medication adherence demonstrate a link between fewer perceived barriers and improved transition readiness, as well as a link between increased adolescent treatment responsibility and improved transition readiness [31]. This suggests that patients who perceive greater challenges or obstacles in the context of their health or self-management, tend to experience more negative attitudes about their preparedness to move through the pediatric to adult care continuum. In contrast, patients who are more engaged in their health management experience more positive attitudes. In combination with our study findings, this is consistent with the empirically supported health beliefs model, whereby perceived barriers to action is a key factor in influencing health behaviors [32].

There are several noteworthy study limitations. First, measures of perceived transition barriers as well as ratings of transition confidence, motivation, importance, and readiness were developed for the purposes of the study and have not yet been validated. However, such factors have repeatedly been shown to lead to greater patient engagement in health care and improved patient outcomes. Second, bias is inherent to any self-report measure, and it is plausible that patients may have endorsed more transition barriers on a screener than they might have during a clinical interview. This was taken into account in the transition barriers survey by including the response choice of “no barriers” and by phrasing the survey instructions as “What, if any, barriers do you think may make it difficult for you to transition into adult care successfully (select all that apply)?”. There is also compelling evidence in the literature that links patients’ self-report of medication adherence barriers to objective health outcomes (e.g., [33,34]). Third, as this study only evaluates patient-reported data, the modified ATR scoring is based on patient reports only. In order to truly determine whether patients are more responsible for IBD-related tasks, patient responses would need to be compared against caregiver reports. Fourth, the finding that perceived barriers did not predict attitudes towards transition may reflect the nature of the barriers being assessed, as most of them tap into the process of transferring from pediatric to adult care rather than engagement in one’s disease management. The same is posited for the weak relationship found between perceived barriers and the allocation of treatment responsibility. To address this, our team is currently furthering our evaluation of perceived barriers towards transition. Our recent work entailed obtaining qualitative feedback from patients and caregivers to develop a comprehensive measure of perceived transition/transfer barriers, and we are currently validating this measure across several pediatric IBD centers in the US, as well as identifying intervention targets that specifically address such perceived barriers. Future endeavors may also include asking patients to rank the order of the barriers, which would provide an opportunity to identity the barriers healthcare providers should prioritize. In addition, while this sample of youth with IBD is demographically similar to the pediatric IBD population at large [35,36], the study findings should be replicated in other pediatric IBD centers, as a more sociodemographically diverse population may have differing attitudes towards transition. The small number of studies in other pediatric populations (e.g., diabetes and sickle cell disease), identifying income and parent education as barriers to transition [37,38], highlights the importance of sociodemographics in shaping one’s transition experience. As such, this study should be replicated among a more socioeconomic and ethnically diverse population, and should also evaluate options to reach patients who lack access to a transition clinic or do not attend such clinic appointments.

## 5. Conclusions

To the best of our knowledge, this study represents the first attempt to summarize patient-reported outcomes that have been collected as part of standard care in a clinic designed to target transition planning, education, and support in pediatric IBD. The study findings will be used to inform the next steps. This will include an evaluation into longitudinal changes in patient-reported outcomes, with repeated visits to the Young Adult IBD Clinic as well as an evaluation into how perceived barriers and attitudes towards transition and transfer impact how patients actually transfer into adult health care systems.

## Figures and Tables

**Table 1:** Participant Characteristics.

Variable	*M* (*SD*) or %
Age (in years)	18.84 (1.26)
Gender (% female)	49.4
Race	
White (non-Hispanic)	71.6
African American	21.0
Hispanic/Latino	3.7
Asian	0.0
Multiracial	3.7
IBD diagnosis	
Crohn’s disease	80.2
Ulcerative colitis	14.8
Indeterminate colitis	4.9
Grade	
11th grade	9.9
12th grade	39.5
College	49.4
Other (e.g., working)	1.2
Insurance type	
Private/Commercial	70.4
Public	24.7
Self-pay	4.9

**Table 2:** Descriptive data for the study measures.

Variable	Mean	Std Dev	Min	Max
Attitude toward transition				
Confidence	7.94	2.00	3.20	10.00
Motivation	7.61	2.40	0.00	10.00
Importance	9.08	1.29	4.90	10.00
Readiness	7.74	2.16	0.00	10.00
Total Score	80.81	17.29	24.75	100.00
Attitude toward transfer				
Confidence	7.30	2.49	0.00	10.00
Motivation	7.29	2.34	0.00	10.00
Importance	8.86	1.50	4.10	10.00
Readiness	7.06	2.58	0.00	10.00
Total score	76.27	19.52	13.00	100.00
Allocation of treatment responsibility				
Medications	−6.35	9.60	−21.00	17.00
Appointments	−0.55	11.71	−21.00	21.00
Labs	−0.32	5.82	−9.00	9.00
Total score	−9.51	24.29	−54.00	40.00
Perceived barriers	3.44	2.21	1.00	10.00
IBD self-efficacy	52.52	6.79	34.00	65.00

**Table 3:** Bivariate Correlations Between Study Variables.

	Age	Transition Attitudes					Transfer Attitudes					ATR				IBD-SES	Barr
		Conf	Mot	Imp	Ready	Total	Conf	Mot	Imp	Ready	Total	Meds	Appts	Labs	Total		
Age	1.00																
Transition																	
*Conf*	0.20	1.00															
*Mot*	0.17	0.78	1.00														
*Imp*	0.31	0.48	0.48	1.00													
*Ready*	0.30	0.83	0.82	0.49	1.00												
*Total*	0.27	0.91	0.92	0.64	0.94	1.00											
Transfer																	
*Conf*	0.12	0.48	0.49	0.03 ^#^	0.50	0.48	1.00										
*Mot*	0.20	0.47	0.57	0.11 ^#^	0.52	0.53	0.81	1.00									
*Imp*	0.18	0.46	0.47	0.51	0.45	0.52	0.42	0.48	1.00								
*Ready*	0.17	0.53	0.53	0.22 ^^^	0.54	0.55	0.82	0.82	0.51	1.00							
*Total*	0.19	0.56	0.59	0.21 ^#^	0.58	0.59	0.92	0.92	0.64	0.94	1.00						
ATR																	
*Meds*	−0.36	−0.42	−0.41	−0.29	−0.43	−0.46	−0.41	−0.32	−0.26 ^#^	−0.35	−0.39	1.00					
*Appts*	−0.52	−0.48	−0.28	−0.33	−0.37	−0.41	−0.26 ^#^	−0.23 ^#^	−0.15 ^^^	−0.26 ^#^	−0.27 ^#^	0.62	1.00				
*Labs*	−0.48	−0.43	−0.28	−0.33	−0.38	−0.40	−0.21 ^^^	−0.19 ^^^	−0.19 ^^^	−0.19 ^^^	−0.22 ^#^	0.47	0.83	1.00			
*Total*	−0.51	−0.49	−0.36	−0.36	−0.44	−0.47	−0.34	−0.28	−0.24 ^#^	−0.32	−0.34	0.84	0.94	0.83	1.00		
IBD-SES	0.14	0.36	0.36	0.29	0.35	0.39	0.23	0.20	0.38	0.26	0.29	−0.23	−0.18	−0.19	−0.20	1.00	
Barriers	0.16	−0.15	−0.12	0.13	−0.10	−0.10	−0.52	−0.36	−0.06	−0.38	−0.41	0.10	0.00	−0.07	0.01	−0.18	1.00

Note: ATR = Allocation of Treatment Responsibility; SES = IBD Self-Efficacy; Conf = Confidence; Mot = Motivation; Imp = Importance; Ready = Readiness; Barr = Barriers. All correlations *p* < 0.01 except ^#^ = 0.01 < *p*< 0.05, and ^^^ = *p* > 0.05.

**Table 4:** Model 1 results: attitudes toward transition path model estimates of direct and indirect effects.

Direct Effects	Estimate	Std Estimate	CI LL	CI UL			
ATR Meds On							
Self-Efficacy	−0.11	−0.08	−0.33	0.20			
Attitude toward Transition	−0.24	−0.43	−0.61	−0.23			
Perceived Barriers	0.26	0.06	−0.19	0.29			
ATR Appts On							
Self-Efficacy	−0.06	−0.03	−0.26	0.19			
Attitude toward Transition	−0.27	−0.40	−0.60	−0.20			
Perceived Barriers	−0.26	−0.05	−0.27	0.16			
ATR Labs On							
Self-Efficacy	−0.06	−0.07	−0.28	0.12			
Attitude toward Transition	−0.12	−0.36	−0.57	−0.15			
Perceived Barriers	−0.32	−0.12	−0.30	0.06			
Self-Efficacy On							
Attitude toward Transition	0.15	0.38	0.18	0.55			
Perceived Barriers	−0.43	−0.14	−0.34	0.07			
Attitude toward Transition On							
Perceived Barriers	−0.78	−0.10	−0.31	0.13			
**Total Indirect Effects**	**Estimate**	**Std Estimate**	**CI LL**	**CI UL**	**Effect Size**	**CI Lower**	**CI Upper**
Barriers to ATR Meds	0.25	0.06	−0.05	0.17	0.02	−0.02	0.06
Barriers to ATR Appointments	0.24	0.05	−0.05	0.15	0.04	−0.05	0.14
Barriers to ATR Labs	0.13	0.05	−0.04	0.16	0.01	0.00	0.02

Note. LL = lower limit; UL = upper limit; CI = confidence interval.

**Table 5:** Model 2 results: attitudes toward transfer path model estimates of direct and indirect effects.

Direct Effects	Estimate	Std Estimate	CI Lower	CI Upper			
ATR Meds On							
Self-Efficacy	−0.22	−0.16	−0.39	0.10			
Attitude toward Transfer	−0.19	−0.37	−0.63	−0.13			
Perceived Barriers	−0.29	−0.07	−0.28	0.17			
ATR Appts On							
Self-Efficacy	−0.21	−0.12	−0.34	0.10			
Attitude toward Transfer	−0.17	−0.29	−0.55	−0.01			
Perceived Barriers	−0.76	−0.14	−0.33	0.06			
ATR Labs On							
Self-Efficacy	−0.13	−0.15	−0.36	0.05			
Attitude toward Transfer	−0.07	−0.25	−0.47	−0.02			
Perceived Barriers	−0.54	−0.21	−0.38	−0.02			
Self-Efficacy On							
Attitude toward Transfer	0.09	0.26	0.04	0.46			
Perceived Barriers	−0.21	−0.07	−0.29	0.16			
Attitude toward Transfer On							
Perceived Barriers	−3.64	−0.41	−0.60	−0.18			
**Total Indirect Effects**	**Estimate**	**Std Estimate**	**CI Lower**	**CI Upper**	**Effect Size**	**CI Lower**	**CI Upper**
Barriers to ATR Meds	0.79	0.18	0.06	0.36	0.07	0.02	0.14
Barriers to ATR Appts	0.75	0.14	0.04	0.31	0.13	0.04	0.28
Barriers to ATR Labs	0.34	0.13	0.04	0.28	0.01	0.00	0.03

Note. LL = lower limit; UL = upper limit; CI = confidence interval.

## Data Availability

The data presented in this study are available in this article.
